# Japanese spotted fever with post-infectious encephalitis

**DOI:** 10.1016/j.idcr.2022.e01658

**Published:** 2022-12-05

**Authors:** Takafumi Wada, Hitoshi Mori, Kouji Kida, Katsuro Shindo

**Affiliations:** a Department of Neurology, Kurashiki Central Hospital, Japan, 1–1-1 Miwa, Kurashiki, Okayama, 710–8602 Japan; b Virology Section, Department of Health Science, Okayama Prefectural Institute for Environmental Science and Public Health, Japan, 739–1 Uchio, Okayama Minami-ku, Okayama, 701–0298 Japan

**Keywords:** Japanese spotted fever, Encephalitis

## Abstract

Japanese spotted fever (JSF) is a rickettsial disease caused by *Rickettsia japonica*. To the best of our knowledge, there have only been five reported cases of JSF involving the central nervous system. A 74-year-old man was admitted after 1 week of fever and maculopapular rash. JSF was definitively diagnosed by PCR; however, the patient showed mental disturbance and abnormal behavior. After intravenous immunoglobulin, his mental state and behavior improved. The findings of cerebrospinal fluid analysis, electroencephalography, and ^99 m^TcHM-PAO single photon computed emission tomography suggested post-infectious encephalitis. JSF causes post-infectious encephalitis and early treatment is recommended.

## Highlights

• We describe a case of Japanese spotted fever (JSF) with post-infectious encephalitis.
• The symptoms improved after initiating intravenous immunoglobulin.
• Post-infectious encephalitis caused by JSF is rare, but treatable with immunotherapy.

## Introduction

Japanese spotted fever (JSF), initially reported in 1984, is a rickettsial infection caused by *Rickettsia japonica* [1]. *Rickettsia japonica* has been detected in 3 genera and 8 species in Japan [16]. It mainly occurs between April and November along the coast of southwestern and central Japan. JSF is also reported in the other Asian countries [2], [3], [4]. JSF is common among people who step into or live in mountains and farmers [17]. The triad of clinical findings comprises fever, erythema, and eschar. It is sometimes accompanied by severe hepatic impairment and thrombocytopenia. Without early treatment, general condition deteriorates due to multiple organ failure and may lead to coma and death. However, there are few cases in which the cause of impaired consciousness has been identified as inflammation of the central nervous system (CNS). We describe a patient with JSF who presented with mental disturbance and abnormal behavior, which responded to intravenous immunoglobulin (IVIg).

## Case report

**Fig. 1:**
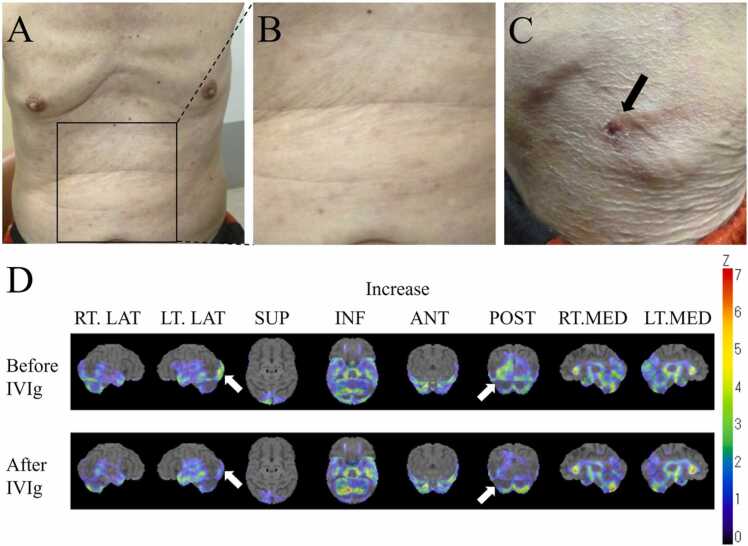
Images of the patient. The patient developed maculopapular rash on the trunk (A, B), back, hip and extremities. Eschar was detected on the left knee (black arrow, C). ^99m^TcHM-PAO single photon computed emission tomography (PAO-SPECT) was performed on the 5th hospital day before initiating intravenous immunoglobulin (IVIg). We detected increased cerebral blood flow in left occipital lobe, using three-dimensional stereotactic surface projection (3D-SSP) analysis (white arrows, D above). On PAO-SPECT performed on the 19th hospital day after the treatment with IVIg, the increased blood flow in left occipital lobe was reduced (white arrows, D below). The both PAO-SPECTs were performed using the following protocol: 1110 MBq of ^99 m^Tc-HMPAO was administered, and imaging was performed on a Dual-head SPECT scanner: Symbia T2 (Siemens Japan) starting about 5 min later. Color coding represents the statistical significance (Z-score) of the increase in regional cerebral blood flow compared with age-matched normal controls. Abbreviations; RT, right; LT, left; LAT, lateral; SUP, superior; INF, inferior; ANT, anterior; POST, posterior; MED, medial.

A 74-year-old male farmer from Okayama Prefecture in Japan was admitted 1 week after the onset of fever and maculopapular rash. He had no history of tick bites. On admission, he was alert, his body temperature was 38.6 °C, systolic blood pressure was 131 mmHg, and pulse rate was 118/min. Lymphadenopathy was not detected. He developed maculopapular rash on the trunk, back, hip, extremities, palms, and soles (Fig. 1A, B). Eschar of 4–5 mm was detected on the left knee (Fig. 1C).

Laboratory examinations revealed a normal white blood cell count (7600 /μL, reference value is 3900–9800 /μL) and a decreased platelet count (83,000/μL, reference value is 131,000–362,000/μL). Aspartate aminotransferase was elevated (107 U/L, reference value is 10–40 U/L), alanine aminotransferase was slightly elevated (45 U/L, reference value is 10–40 U/L), and total bilirubin was elevated (1.6 mg/dL, reference value is 0.3–1.2 mg/dL). Lactate dehydrogenase was nearly normal (303 U/L, reference value is 124–222 U/L). Inflammatory markers (ferritin 982 ng/mL, reference value is 39.4–340 ng/mL and C-reactive protein 11.21 mg/dL, reference value is less than 0.14 mg/dL) were high.

Based on the high fever, maculopapular rash, eschar, and natural exposure (farming), we suspected JSF, and, thus, initiated the oral administration of doxycycline (200 mg/day). JSF was definitively diagnosed by real-time polymerase chain reaction (PCR) analysis of blood and crust. We performed 2 real-time PCR targeting 16 S rDNA and 216 bp ORF developed by Kawamori et al. [5] and Hanaoka et al. [6], respectively. Sequence analysis of the crust showed 100 % identical to the standard strain of *Rickettsia japonica*, YH strain, in part of the genus Rickettsia–specific outer membrane protein 17 kDa gene (DDBJ/ENA/Genbank accession number of 394 nt is **LC661677**) and the citrate synthase gene (*gltA*) (DDBJ/ENA/Genbank accession number of 322 nt is **LC661678**). The doxycycline treatment reduced his body temperature to 36 °C and partially improved maculopapular rash. However, after 4 days of treatment of doxycycline and fever resolution, the patient subsequently became disorientated and exhibited abnormal behavior. He also presented with hallucinations and mental disturbance. A physical examination did not reveal nuchal rigidity. We performed lumbar puncture.

CSF analysis showed a normal white blood cell count of 1 leukocyte /μL (reference value is less than 5 /μL), an almost normal glucose level of 82 mg/dL (reference value is 50–75 mg/dL, blood sugar was 201 mg/dL) and a normal protein level of 40 mg/dL (reference value is 15–45 mg/dL). CSF IL-6 (15.7 pg/mL, reference value is less than 4.0 pg/mL) and IL-8 (165.0 pg/mL, reference value is less 5.4–59.0 pg/mL [7]) levels were elevated. Electroencephalography (EEG) revealed rhythmic delta activities in left posterior temporal region. Brain magnetic resonance imaging was normal. The increased cerebral blood flow in left occipital lobe was detected on ^99 m^TcHM-PAO single photon computed emission tomography (PAO-SPECT), using three-dimensional stereotactic surface projection (3D-SSP) analysis (Fig. 1D).

Based on these findings, post-infectious encephalitis was suspected. IVIg was administered for 5 days (400 mg/kg/day), and his abnormal behavior and hallucination improved over several days. Doxycycline was administered for a total of 8 days. The increased blood flow in left occipital lobe was reduced on PAO-SPECT performed 2 weeks after IVIg (Fig. 1D). He had no neurological sequelae.

## Discussion

Our case was thought to be post-infectious encephalitis caused by JSF. In post-infectious encephalitis, neurological symptoms appear abruptly with a latency period of 2–30 days after infection began, and evolve over hours to days [8], [9]. The patient presented with abnormal behavior, mental disturbance, and hallucination after 4 days of administration of doxycycline and fever resolution. The symptoms improved early after initiating IVIg therapy. It is thought that IVIg rather than doxycycline improved the symptoms and the clinical course was consistent with post-infectious encephalitis. Antibiotic-associated encephalopathy needs to be considered as a differential diagnosis. However, there is currently no evidence to show that doxycycline is associated with encephalopathy [10]. Moreover, his neurological symptoms improved during treating with doxycycline. So, the present case did not appear to be antibiotics-associated encephalopathy.

To the best of our knowledge, there have only been five reported cases of JSF involving the CNS which are described as aseptic meningoencephalitis [11], [12], [13], [14]. Three of them presented with convulsion, and all of them presented with consciousness disturbance or loss of consciousness. All of them had CNS symptoms before the initiation of antibiotics to treat JSF. Imaging study revealed no abnormalities except for one case with subdural hematoma. Our case differed from previous cases because of the presence of abnormal behavior and hallucinations, which seemed to be symptoms of encephalitis.

The levels of CSF IL-6 and IL-8 were characteristically elevated in the present case. Excessive host immunity has been associated with post-infectious encephalitis. The systematic review by Kothur et al. reported that CSF IL-6 and IL-8 levels were elevated in infectious encephalitis and acute disseminated encephalomyelitis [7]. CSF cytokine levels in JSF have not been reported; however, based on the elevation of CSF IL-6 and IL-8 levels, the excessive host immunity was indicated to be involved in the present case.

Moreover, the rhythmic delta activities of left posterior temporal region in the EEG and the changes of cerebral blood flow in left occipital lobe, detected on PAO-SPECT using 3D-SSP analysis, were also consistent with encephalitis. The EEG findings was suggestive of left temporal lobe abnormalities, which may have been associated with the mental disturbance. The increased blood flow was reduced when the symptoms disappeared after IVIg, so it was probably associated with the hallucination.

The present case did not have pleocytosis or an elevated CSF protein level. However, in post-infectious encephalitis, CSF examination can be normal in 19–33% of adult patients [8]. As a limitation, we note that we could not perform CSF PCR of *Rickettsia japonica*; however, CSF PCR of *Rickettsia japonica* has never been performed in the cases of JSF with CNS involvement [11], [12], [13], [14].

Post-infectious encephalitis responds well to steroids and IVIg [15]. The initiation of IVIg on the assumption of acute encephalitis caused by JSF resulted in a good clinical outcome. JSF may cause multiple organ failure or mental disturbance without appropriate treatment, sometimes resulting in death. As described above, few cases involving CNS, such as meningoencephalitis, have been reported to date; however, post-infectious acute encephalitis by JSF may be overlooked. Therefore, physicians should be aware of this rare manifestation of JSF which can be treated with immunotherapy.

## Ethical approval statement

Written informed consent was obtained from the patient for publication of the detail of his medical case and any accompanying images.

## Funding

This research did not receive any specific grant from funding agencies in the public, commercial, or not-for-profit sectors.

## Declarations of interest

The authors declare that there is no conflict of interest.
